# Role of BRCA Mutations in the Modulation of Response to Platinum Therapy

**DOI:** 10.3389/fonc.2018.00016

**Published:** 2018-02-05

**Authors:** Sanghamitra Mylavarapu, Asmita Das, Monideepa Roy

**Affiliations:** ^1^Invictus Oncology Pvt. Ltd., Delhi, India; ^2^Department of Biotechnology, Delhi Technological University, Delhi, India

**Keywords:** breast cancer, ovarian cancer, BRCA1/2 mutations, platinum drugs, response, resistance

## Abstract

Recent years have seen cancer emerge as one of the leading cause of mortality worldwide with breast cancer being the second most common cause of death among women. Individuals harboring BRCA mutations are at a higher risk of developing breast and/or ovarian cancers. This risk is much greater in the presence of germline mutations. BRCA1 and BRCA2 play crucial role in the DNA damage response and repair pathway, a function that is critical in preserving the integrity of the genome. Mutations that interfere with normal cellular function of BRCA not only lead to onset and progression of cancer but also modulate therapy outcome of treatment with platinum drugs. In this review, we discuss the structural and functional impact of some of the prevalent BRCA mutations in breast and ovarian cancers and their role in platinum therapy response. Understanding the response of platinum drugs in the context of BRCA mutations may contribute toward developing better therapeutics that can improve survival and quality of life of patients.

## Introduction

Cancer is one of the leading causes of mortality worldwide. As per WHO estimates, 8.8 million cancer related deaths were reported in 2015, and this number is projected to rise to 13.1 million by the year 2030, with low- and middle-income countries bearing approximately 70% burden of all deaths worldwide. Of these, breast cancer has emerged as the second major cancer type comprising almost 25% of all cancers among women. Breast cancer is the most commonly occurring cancer among women with incidence rates varying widely across the world, having rates ranging from 27 per 100,000 in Middle Africa and Eastern Asia to 92 in Northern America. But the mortality rate is lower in the developed countries as compared with low- and middle-income countries, because of higher survival of breast cancer patients in developed nations (1, 2).

Mutations in breast cancer susceptibility gene type 1 and type 2 (BRCA1 and BRCA2) put women at a higher risk of developing breast and/or ovarian cancer. In individuals harboring mutations in BRCA1, the probability of developing breast cancer over a lifetime is about 57–65% and that of ovarian cancer is about 39–40%. With BRCA2 mutations, the probabilities are at 45–49% for breast cancer and 11–18% for ovarian cancer (3, 4). Women with germline mutations are more prone to develop these cancers at a younger age with more aggressive disease and poorer prognosis as compared to those with somatic mutations. BRCA-mutated tumors exhibit both higher clinical grade and stage disease with greater metastatic potential (5). 70% of breast tumors containing germline mutations in BRCA1 fall in the category of triple-negative breast cancer (TNBC), a highly aggressive, highly metastatic subtype, comprising approximately 15% of all breast cancer cases, characterized by the absence of hormone receptors with no amplification of growth signal receptor (6).

Ovarian cancer patients with BRCA mutations exhibit a higher histological grade disease compared to those with sporadic disease and respond better to platinum therapy, having better prognosis (7, 8). In the absence of functional BRCA proteins, cells fail to repair intra-strand crosslinks formed by DNA cross-linking agents such as platinum drugs, leading to apoptotic cell death. Cisplatin is the most commonly used therapeutic agent for treating gynecological cancers either as a single agent before surgery or in combination with other drugs. Despite the favorable initial response, these cancers eventually develop tolerance to platinum leading to therapy failure. This review examines a few clinically relevant mutations that are common in breast and ovarian cancers. The structural and functional changes resulting from these mutations are explored further, focusing on their implications in modulating response to platinum therapy.

## BRCA Mutations in Breast and Ovarian Cancers

BRCA1 and BRCA2 play a crucial role in maintaining genome integrity by repairing double-strand DNA breaks *via* the homologous recombination repair (HRR) pathway. Any mutations that cause functional disruption of these proteins may prove to be highly deleterious, leading to the development of cancer. In addition, BRCA1 and BRCA2 also play a critical role in cell division where they are transported to the cytosol to participate in regulating various molecular events during mitosis. Mutations impacting these important functions of BRCA1/2 can affect the delicate balance of the tightly regulated cellular processes that may lead to progression of disease.

BRCA mutations show huge diversity in various populations, many of which are functionally neutral or are of unknown pathological significance. However, there are some mutations that are more significant than others (Table 1). One of the most common cancer related mutations found in BRCA1 is the 5382insC, reported to have originated from a common European ancestor about 400–500 years ago. It was first described as the founder mutation in the Ashkenazi Jew population and could be present in other European populations as well. This mutation is also reported to be associated with a higher incidence of ovarian cancer (9.4%) but a lower incidence of breast cancer in Slavic countries (9). 185delAG located in exon 2 of BRCA1 is another common mutation reported in various ethnicities including Ashkenazi Jews and Indian population where it occurs at a high frequency of 16.4%. Missense mutation at the Cys61 (C61G) of BRCA1 is a founder mutation in Polish population and is included as a standard test for diagnosis and treatment of breast and ovarian cancer for Polish women (10, 11). 6174delT mutation is common in BRCA2 in the Ashkenazi Jewish population and other ethnic groups (12). In addition, BRCA1 and BRCA2 contain numerous other mutations that show a more population-specific distribution. This has been summarized in a review by Karami and Mehdipour (9). Screening for specific BRCA1/2 mutations that occur at high frequency in certain populations not only help in better clinical management of breast and ovarian cancers but can also be an invaluable tool in identifying healthy individuals who are currently disease-free but are at an increased risk of developing breast and/or ovarian cancer later in life (13). In addition to the presence of either BRCA1 or BRCA2 mutations in breast and/ovarian cancer patients, there are reports of patients being double heterozygous for both BRCA1 and BRCA2 mutations. These patients develop cancer at a much earlier age and with more severe disease (14, 15).

**Table 1:** Clinically significant BRCA1/2 mutations in breast and ovarian cancers.

Type of mutation	Amino acid position	Amino acid change	Functional significance
**BRCA1**			

Missense	10	E → K	Familial breast and ovarian cancer
	23	E → K	Familial breast and ovarian cancer
	61	E → K	Breast and ovarian cancer. Interaction with BAP1 lost
	64	C → G	Loss of interaction with BAP1 in breast cancer
	67	D → Y	Breast cancer. Decreased ubiquitin function of BRCA1
	1685	T → I	Could be associated with susceptibility to cancer
	1699	R → Q	Reduced affinity for BRIP1 phosphopeptide in breast cancer
		R → W	Reduced protein stability breast and ovarian cancer
	1749	P → R	Reduced binding to BRIP1
	1775	M → K	Breast cancer. Interaction with BRIP1 and RBBP8 lost
Deletions	185delAG		Exon 2. Truncated protein. Functional null
	Δ369		Deleted in breast cancer
Insertion	5382insC		Breast and ovarian cancer. C-terminal truncated protein

**BRCA2**			

Missense	25	G → R	Breast cancer. PALB2 interaction lost
	31	W → C/R	Breast cancer. PALB2 interaction lost
	372	N → H	Common polymorphism that may elevate the risk of breast cancer
	Δ1286		Deleted in breast cancer
	Δ1302		Deleted in breast cancer
	2336	R → H	Decreased homologous recombination repair
	2722	T → R	Breast cancer. Exon skipping resulting in out of frame exons 17 and 19 fusion
	2723	D → H	Promotes RAD51 cytoplasmic localization in heterozygous state
Frameshift	6174delT		Truncated protein. BRCA1 C-terminus domain, NLS lost. Rad51 interaction lost

## Cancer-Associated Mutations Alter the Biology of BRCA Proteins

**Figure 1:**
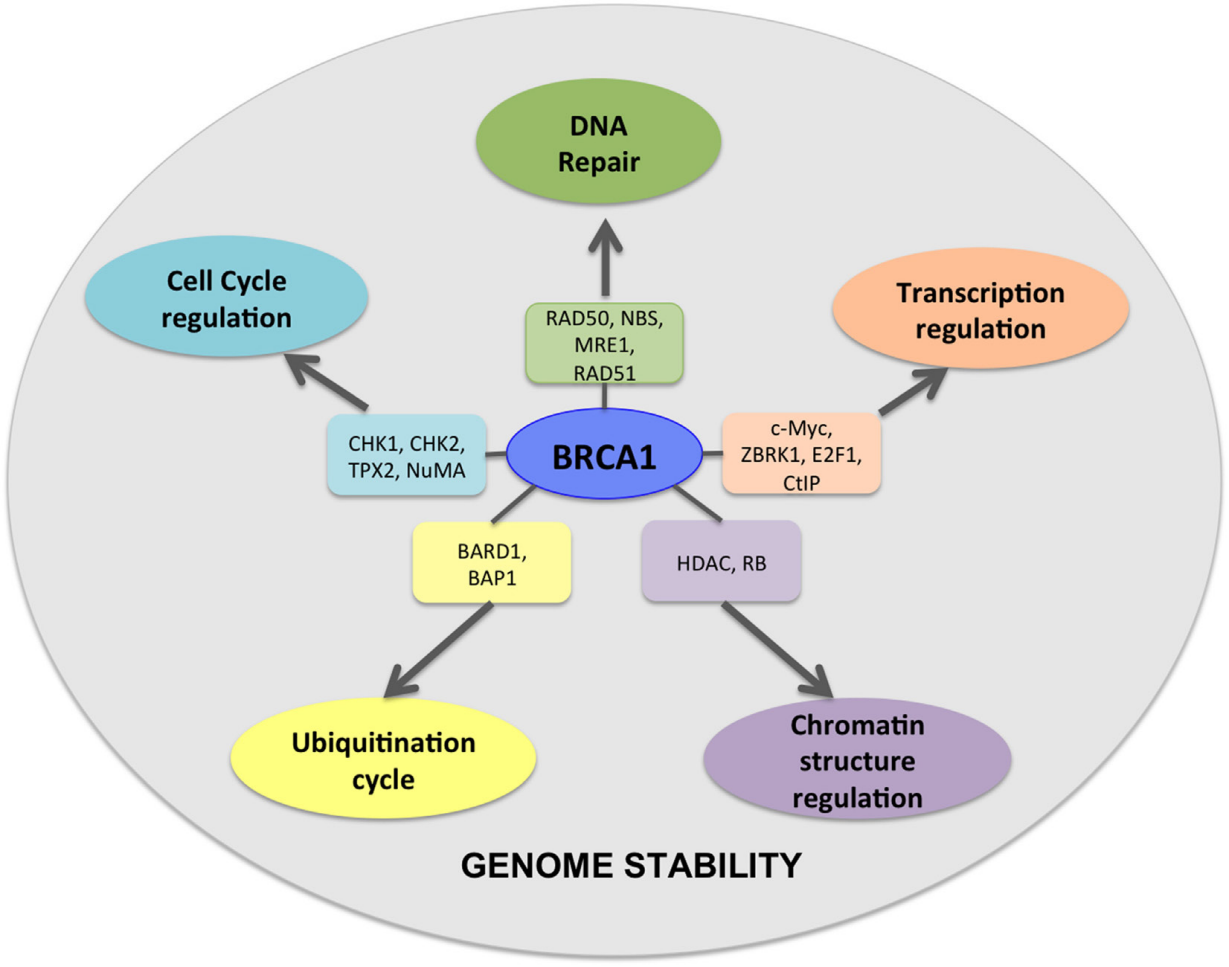
BRCA1 is at the hub of numerous interconnecting cellular pathways. BRCA1 interacts with numerous intermediate proteins in these pathways that contribute toward genomic and cellular stability.

BRCA1 and BRCA2 are at the intersection of numerous key cellular pathways and perform multiple functions ranging from DNA damage response and DNA repair activity, chromatin remodeling and transcription, and protein ubiquitination (16–19) (Figure 1). Single- or double-strand DNA breaks may arise from various sources like natural metabolic processes or from extraneous sources like chemical agents or irradiation. Left unrepaired, these DNA breaks may lead to the accumulation of deleterious mutations with a potential to cause genomic instability. Efficient repair of double-strand breaks *via* the HRR pathway requires functional involvement of BRCA1 and BRCA2 *via* their interaction with numerous other proteins (20, 21). Upon DNA damage, BRCA1 acts as a mediator bringing together components of the DNA repair pathway to the site of damage where it interacts with a large complex called the BRCA1-associated genome surveillance complex (BASC complex), along with the components of the DNA repair machinery (22, 23). Although BRCA1 and BRCA2 function in the DNA damage repair pathway, both have functionally distinct roles (23). BRCA1 functions as a DNA damage checkpoint activator and also in DNA repair, whereas BRCA2 is a core component of the HRR machinery. Homozygous BRCA1 knockout mice are embryonic lethal at age E7.5–E13.5, suggesting that functional loss of BRCA1 cannot be compensated by the presence of wild-type BRCA2 (24).

**Figure 2:**
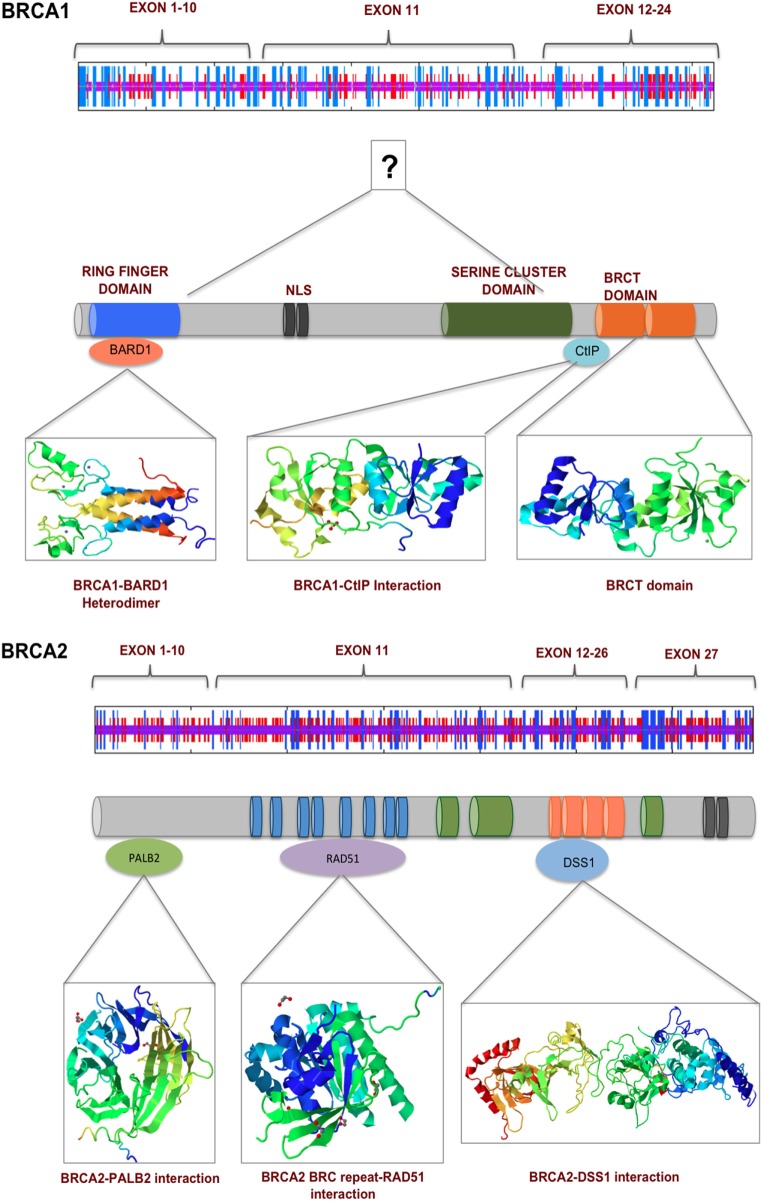
BRCA1 and BRCA2 interact with numerous proteins *via* their multiple functional domains. The N and C termini of BRCA1 have structural motifs that allow multiple protein–protein interaction. Exons 11–13 in the middle of BRCA1 are more unstructured, which contains two nuclear localization signals. BRCA2 secondary structure prediction indicate a more helical middle region, which contains BRC repeats and binding sited for various proteins of the DNA damage response pathway.

**Figure 3:**
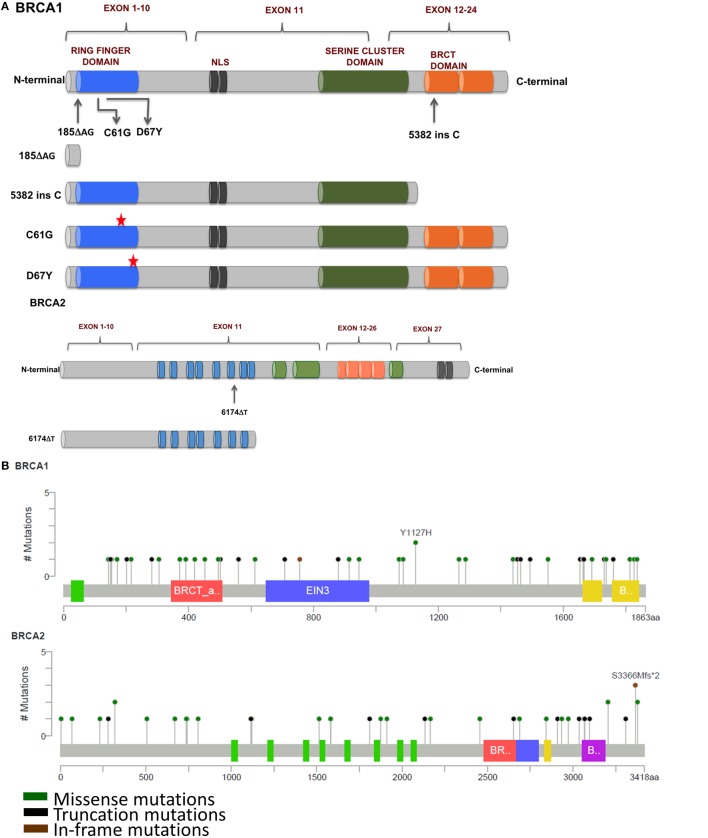
**(A)** Mutations in BRCA1 and BRCA2 found in breast and ovarian cancers that affect response to platinum therapy. Deletion of multiple functional domains from BRCA1 result in truncated protein that is unable to repair DNA damage efficiently whereas missense mutations that result in amino acid substitutions result in altered response to platinum therapy. **(B)** Schematic representation of prevalent somatic mutations in BRCA1 and BRCA2 from breast cancer patients showing relative positions of various missense, truncations, and in-frame mutations (Adapted from www.cBioPortal.org) (31).

The BRCA proteins are organized into functional domains that enable numerous protein–protein interactions that are vital for their optimal function. BRCA1, a 220 kDa protein, contains an RING domain in the N-terminus that interacts with BRCA1-associated RING domain protein 1 (BARD1), the heterodimerization of which increases BRCA1 ubiquitin ligase activity by many folds. The BRCA1 C-terminus domain is conserved across many proteins that are involved in DNA repair and is the site for numerous phosphoprotein interactions. Limited structural data are available for the region of BRCA1 between exons 11 and 13, despite it being the binding site for a number of proteins that are involved in multiple cellular pathways. Some of the proteins that bind to this region of BRCA1 are retinoblastoma protein (RB), the transcription factor c-Myc, DNA repair proteins RAD50, RAD51, and PALB2 that forms a scaffold for BRCA1 and BRCA2 interaction (25). A common feature shared by many proteins that are at the hub of interconnecting pathways is the intrinsically disordered structure (26). Exons 11–13 of BRCA1 exhibit such disordered structure that perhaps provide a scaffold for multiple interactions and signal integration from various pathways (27). BRCA2 is a large 385 kDa protein with an N-terminus transactivation domain, a long exon 11 containing RAD51-specific binding site and a DNA binding domain toward the C-terminus (28). BRCA2, like BRCA1, contains disordered structure interspersed between more structured motifs, suggesting its participation in multiple cellular pathways (29, 30) (Figure 2). Here, we discuss some key mutations in BRCA1 and BRCA2 that have strong correlation with breast and ovarian cancer and their functional consequences (Figures 3A,B).

*185delAG*—A two nucleotide deletion in exon 2 of BRCA1 produces a 39 amino acids long, functionally null truncated protein that has lost most of its functional domains as compared with a 220 kDa wild-type protein. The loss of the ring finger domain abrogates the binding of BARD1 and BAP1, which are important regulators of E3 ubiquitin ligase activity of BRCA1. Therefore, the loss of the ring finger domain of BRCA1 and consequently, loss of E3 ubiquitin ligase activity may be an important event in the development and progression of breast cancer (32, 33). BRCA1 interacts with both upstream and downstream effectors of cell cycle checkpoint kinases—deletion of binding domains result in the loss of these interactions and failure of the S and G2/M phase checkpoints allowing cell cycle progression even in the presence of DNA damage (34–37). Interaction of BRCA1 and BRCA2 is mediated by PALB2, and the complex is critical in RAD51-mediated HRR of damaged DNA. Disruption of this interaction by functional loss of BRCA1 protein results in defective DNA repair and consequently propagation of mutations that accumulate in daughter cells during cell division (38). However, nuclear expression of Maspin (mammary serine protease inhibitor), a member of the serpin superfamily and a target of truncated BRCA1, has been correlated with increased sensitivity to cisplatin with improved prognosis in ovarian cancer (39).

*5382insC* produces a C-terminal truncated BRCA1 commonly encountered in breast and ovarian tumors. The C-terminus of BRCA1 contains numerous domains for various protein–protein interactions including RNA-helicase binding, HDAC interaction, CtIP binding, and many others proteins (40–42). Thangaraju and colleagues have reported that the deletion of the C-terminus of BRCA1 through the 5382insC mutation resulted in loss of apoptosis in cell lines. It has been speculated that the deletion of the transactivation domains may be responsible for loss of transcriptional activation and/or repression of several genes, which may lead to apoptotic cell death (43).

*6174delT*—A frameshift mutation in the BRCA2 produces a 224 kDa truncated protein, about 2,002 amino acids long as compared with the 3,418 amino acids long wild-type protein (390 kDa). This truncation leads to the loss of two BRC repeat domains, loss of DNA binding, and the C-terminus RAD51 binding domain along with the nuclear localization signal, resulting in cells’ defective DNA repair machinery that are unable to form RAD51 foci (44). In addition, cells carrying this mutation have a higher sensitivity to inhibitors of poly(ADP-ribose) polymerase (PARP). Edwards et al. reported that deletion of the 6174delT mutation could make the otherwise PARP inhibitor sensitive cells resistant to the drug. This acquired resistance to PARP inhibitors could be reversed upon restoration of the reading frame of BRCA2 (45), providing a window of opportunity for development of newer and effective strategies for clinical management of BRCA2-mutated cancers. C-terminal truncation of BRCA2 results in the loss of DSS1-mediated stabilization and rapid degradation of BRCA2, leaving cells vulnerable to DNA damage (46, 47). Other interacting partners of BRCA2 include EMSY and DMC1. Sporadic breast and ovarian tumors show amplification of EMSY, a binding partner of BRCA2, and have been associated with poor survival. Interaction of EMSY with exon 3 of wild-type BRCA2 (deleted in cancer) functionally inactivates BRCA2, suggesting a regulatory role for EMSY in the HRR pathway (48). The region between amino acids 2386 and 2411 of BRCA2 is highly conserved across species and is the binding site for DMC1, a germ cell counterpart of RAD51. Loss of this interaction due to the 6174delT mutation results in defective meiosis and propagation of chromosomal abnormalities in the germline (49).

## Sporadic Cancer and BRCAness

BRCA1/2 mutations, seen most commonly in familial breast and ovarian tumors, impact the DNA repair pathway leading to genomic instability. However, some sporadic tumors that contain wild-type BRCA1 also have defective DNA repair pathway that may have resulted *via* other mechanisms. These characteristics of sporadic tumors that are similar to familial cancers are collectively called “BRCAness.” Inactivation of BRCA1 in sporadic breast and ovarian tumors may be brought about by non-genetic mechanisms like promoter methylation that result in lowering of gene expression to undetectable levels and loss of heterozygosity. In contrast, BRCA2 inactivation does not occur by promoter hypermethylation—a significant number of sporadic breast and ovarian tumors show amplification of EMSY at the gene level. As discussed earlier, EMSY–BRCA2 interaction may regulate DNA repair *via* HRR pathway (50). A common feature that both BRCA-mutated cancers and those showing characteristics of BRCAness share is the elevated susceptibility to DNA cross-linking agents like platinum drugs and this has been the rationale for including these as therapeutic agents (8).

## BRCA-Mutated Tumors are Sensitive to Platinum Therapy

Ever since its approval as an antineoplastic agent, cisplatin has been effective in BRCA-mutated breast cancer either as a single agent or in combination with other anticancer drugs (51, 52). The involvement of BRCA1 in efficient DNA repair mechanism has been highlighted by *in vitro* studies that showed that cells containing mutant BRCA1 showed increased sensitivity to platinum drugs as compared with those cells that have elevated BRCA1 levels. This heightened sensitivity could, however be, reversed upon restoration of the full-length functional BRCA1 (53, 54). BRCA1 has been identified as a key gene of the DNA repair machinery not only by siRNA screens but also using BRCA1, TP53 conditional knockout mice where animals after an initial favorable response to cisplatin, doxorubicin, and docetaxel, became resistant to doxorubicin and docetaxel but remained sensitive to cisplatin (55). Breast cancer xenografts using HCC1935 with mutated BRCA1 showed complete inhibition of tumor growth upon treatment with cisplatin whereas only partial response in xenografts that had the wild-type BRCA1 reconstituted. Moreover, significant cell cycle arrest at the S phase and G2/M transition was observed. This not only points to the role of BRCA1 in DNA repair pathway and cell cycle checkpoint activation but also its involvement in modulating response to platinum drugs (56, 57).

Although BRCA-mutated breast tumors exhibit higher histological grade due to increased accumulation of chromosomal aberrations over time and are particularly sensitive to platinum drugs, their therapeutic use is limited to first-line treatment mainly due to development of resistance (58–62). This was demonstrated as early as 1988 when 47% partial response was achieved with a dose of 30 mg/m^2^/day of cisplatin for 4 days, every 3 weeks, in six cycles as a first-line therapy for metastatic breast cancer (63). In another study, 83% pathologic complete response (pCR) was reported in breast cancer patients treated with cisplatin as a neoadjuvant treatment for TNBC. Also, higher response to platinum and pCR has been shown to be associated with low BRCA1 expression, promoter methylation, p53 frameshift or nonsense mutation, and E2F3 activation (64). Huang et al. reported more than 11 years of remission in a BRCA2-mutated metastatic breast cancer patient who received chemotherapy using high dose of cisplatin along with anthracyclin and alkylating agents (65). BRCA1-mutated metastatic breast cancer that was unresponsive to docetaxel, responded well to cisplatin and gemcitabine combination therapy for more than 6 months (66). In case of ovarian cancer, BRCA1 and BRCA2 patients exhibit a differential age of tumor development—BRCA1 carriers develop tumors earlier than those carrying BRCA2 mutations (48 vs. 57 years). Also, BRCA mutations seem to render the tumors more responsive to platinum drugs with a better survival (91 vs. 54 months) and longer disease-free interval (49 vs. 19 months) as compared with sporadic ovarian cancer (67, 68).

## Secondary Somatic BRCA Mutations Contribute to the Development of Resistance to Platinum Drugs

28.3% of recurrent ovarian cancer tumors contain secondary mutations as compared with only 3.1% in primary tumors, while 46.2% of platinum resistant tumors have secondary mutations that restored the function of BRCA1/2 as compared with 5.3% that are sensitive to platinum drugs (69). This implies that the secondary mutations in BRCA1/2 may be instrumental in the development of drug resistance to platinum therapeutics. The 39 amino acid long, non-functional BRCA1, produced due to the 185delAG mutation in the exon 2, severely impair its DNA repair function making the cells highly sensitive to platinum. This may get circumvented by the restoration of the reading frame that produces the full-length functional protein resulting in efficient DNA damage repair. Various studies indicate that platinum refractory BRCA1-mutated tumors carry two nucleotide insertions that are otherwise not present in BRCA1-mutated platinum-sensitive tumors. Similar results were demonstrated in case of BRCA2 mutations. In addition, new secondary mutations arise due to selection pressure exerted by prolonged exposure to platinum drugs resulting in resistance to both platinum drugs as well as PARP inhibitors (70). Such accumulation of secondary mutations could contribute to the development of resistance to platinum drugs over time. The presence of secondary mutations restoring the function of BRCA1/2 could be developed as a promising prognostic marker for either screening patients that are likely to respond favorably to platinum therapy or for predicting clinical outcome in patients already receiving platinum drugs or PARP inhibitor as single agent or in combination (71).

Apart from accumulated secondary mutations in BRCA, missense mutations in the ring finger domain affect response to platinum drugs. C61G missense mutation in exon 5 of BRCA1 abrogates BRCA1–BARD1 heterodimerization leading to loss of E3 ubiquitin ligase activity and cytoplasmic mislocalization of BRCA1. This results in diminished availability of BRCA1 at the site of DNA damage and impaired DNA repair, increasing vulnerability of these cells to platinum drugs. Homozygous C61G mice are embryonic lethal with severe developmental delays, mimicking phenotype of BRCA1 null mice. Interestingly, unlike BRCA1 null mice, these mice were reported to have developed resistance to both cisplatin and the PARP inhibitor olaparib, suggesting a hypomorphic activity of C61G mutation in BRCA1 (72). D67Y, another missense mutation that substitutes aspartic acid for a tyrosine residue at the 67th position result in decreased ubiquitin function of the mutated BRCA1 protein. In contrast to the C61G mutation, the D67Y mutation has been reported to increase cisplatin sensitivity *in vitro* (73).

**Figure 4:**
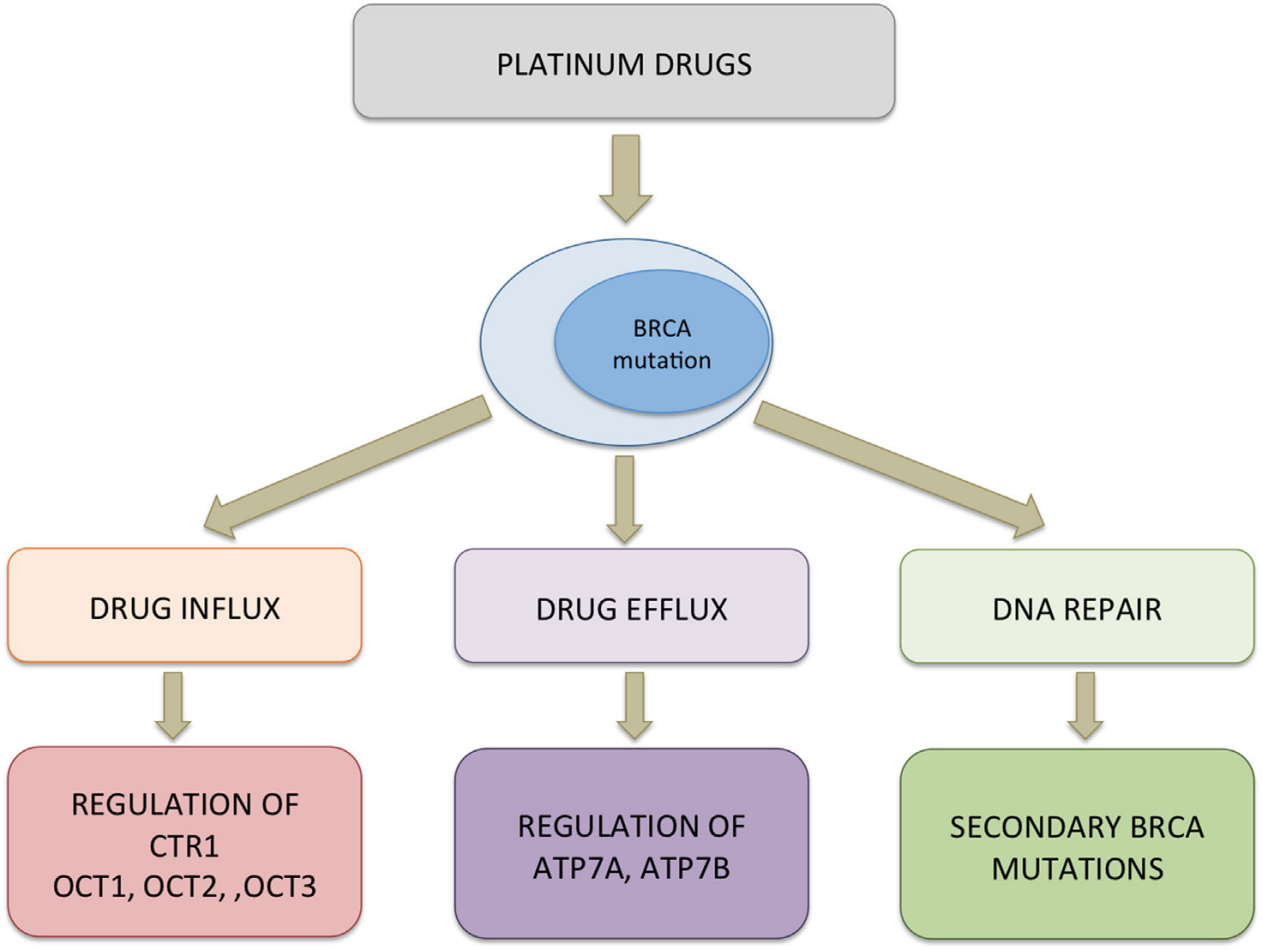
Probable mechanism of resistance to platinum drugs in BRCA-mutated cells. Multiple pathways could be activated simultaneously conferring resistance to platinum drugs in cells. Downregulation of copper transporter (CTR1) and organic cation transporters (OCT1/2/3) can decrease the uptake of platinum drugs whereas upregulation of efflux pumps can bring about increased transport of the drug out of the cells. In the cytosol, platinum drugs can bind to a protein such as chaperons that interfere with the drug reaching the target. Functional restoration of BRCA1/2 and elevated expression of ERCC1 leading to effective removal of platinum–DNA adducts and efficient DNA repair contribute to decreased cytotoxicity of platinum drugs.

In addition to mutational inactivation of BRCA function contributing to resistance to platinum drugs, several other mechanisms may exist that result in tumors not responding to platinum therapy (Figure 4). The dynamic balance between influx and efflux of platinum drugs influence its availability in the intracellular milieu to form DNA adducts and ultimately lead to apoptotic cell death. Copper transporters (CTR1 and CTR2) and copper-transporting p-type adenosine triphosphatase 1 and 2 (ATP7A and ATP7B) are key molecules that are responsible for intracellular transport of platinum drugs. In a meta-analysis study, Sun and colleagues reported positive correlation between CTR1 expression levels and response to cisplatin with favorable overall survival, progression, and disease-free survival. However, no such correlation could be made for CTR2, ATP7A, and ATP7B (74). Therefore, how a patient would response to platinum therapy is dependent not only on the occurrence of mutations that functionally inactivate BRCA1/2 but also to a large degree on other cellular mechanisms such as ion transport pathways that influence the dynamics of the drug within the tumor.

## Overcoming Platinum Resistance in BRCA-Mutated Cancers

Treatment failure as a consequence of development of resistance to platinum drugs resulting in disease recurrence is a major roadblock in clinical management of cancers that carry BRCA mutations. In such cases, non-platinum-based drugs can achieve improved response and extended survival. PARP-1 and PARP-2, members of the PARP family, recruit the base excision repair (BER) machinery, a parallel, but less efficient mechanism for repairing damaged DNA, to the site of single-strand DNA breaks. In the absence of PARP, these lesions persist resulting in stalling of the replication fork during DNA synthesis and formation of DSBs (75). The concept of synthetic lethality, the underlying premise of which is that functional depletion of two genes singly may not cause deleterious effect but together are lethal, was thus introduced to cancer therapeutics to develop better treatment strategies. This was mainly achieved by blocking the BER pathway by small molecule inhibitors against PARP in BRCA1/2-mutated cancers (76).

Poly(ADP-ribose) polymerase inhibitor olaparib has recently received FDA approval for treatment of ovarian cancer with mutated BRCA1/2 and EMEA approval for maintenance therapy for platinum-sensitive ovarian cancer (77). Randomized phase II clinical trial for evaluating the efficacy of olaparib, the first clinically approved PARP inhibitor, in combination with either paclitaxel or carboplatin, followed by olaparib monotherapy resulted in improved progression-free survival in BRCA-mutated high grade ovarian cancer patients compared with treatment with paclitaxel and carboplatin (78). PARP inhibitors have also been reported to be effective in other BRCA-mutated cancers. Clinical trials are underway to assess the efficacy of these (79, 80). Other PARP inhibitors such as niraparib, veliparib, and rucaparib are currently being tested in various clinical trials (81). Kim et al. reported that there is no clear correlation between BRCA1 expression and response to docetaxel (82), while Byrski et al. reported that non-BRCA1 mutation carriers showed higher complete or partial response to docetaxel as neoadjuvant therapy when compared with patients who were BRCA1 mutation carriers (83). *In vitro* studies using breast cancer cells indicate that BRCA1 mutations make the cells non-responsive to taxanes, an observation supported by *in vivo* experiments where targeted deletion of p53 and BRCA1 in mammary tissue rendered tumors resistant to docetaxel but not cisplatin (52). Clinical correlation between BRCA1 mutation and response to taxane is not clear with several studies reporting data that are largely inconclusive.

## Conclusion

Structural organization of BRCA1 and BRCA2 into functionally distinct domains allow for multiple protein–protein interactions with numerous binding partners that facilitate participation in various cellular activities including DNA damage repair pathway. Specific protein–protein interaction such as BRCA1 and BARD1 that is crucial for the E3 ubiquitin ligase activity and the numerous phosphoproteins that bind to the C-terminus of BRCA1 make it an important node in the highly branched intracellular signaling network (29, 30, 84–88). Most common type of mutation observed among breast and ovarian cancer patients is deletion of multiple functional domains of BRCA1/2 that lead to not only impaired DNA repair but also abrogation of cell cycle checkpoints and transcriptional mis-regulation of genes, which eventually lead to global genomic instability. Suboptimal DNA repair mechanism arising from BRCA mutations render cells highly vulnerable to DNA cross-linking agents, such as platinum drugs, making them useful therapeutics in many cancers. *In vitro* data have provided strong evidence toward better response to platinum drugs in the presence of BRCA mutations and is corroborated by clinical studies where BRCA mutation carriers exhibit better survival and longer disease-free intervals upon treatment with platinum drugs, suggesting beneficial therapy outcome. Along with targeting the suboptimal HRR pathway that results from non-functional BRCA1/2, the blocking of the BER pathway with PARP inhibitors significantly improves survival rates of cancer patients. Although BRCA1/2-mutated tumors are highly susceptible to platinum drugs and PARP inhibitors, development of resistance poses a major challenge in the clinical management of these cancers and secondary mutations significantly contribute toward this.

The inherent susceptibility of BRCA-mutated tumors to platinum drugs makes it an appropriate target for development of newer therapeutic agents. The ongoing efforts to design and develop novel inhibitors for the various components of the DNA repair pathway may yield encouraging results and in combination with platinum drugs could further improve the treatment options available for cancer patients. In addition, molecular signatures that can predict the outcome of a treatment regimen are being evaluated as biomarkers, which may help in identifying a target population that is more likely to respond to therapy. For instance, the C118T and C8092A polymorphisms in ERCC1 have been strongly correlated with objective response rate and overall survival (89). Similarly, the levels of annexin A3 in the peripheral blood may be a potential predictor for platinum resistance in ovarian cancer (90). Although molecular markers have the potential to predict therapy response, rigorous validation in large patient cohorts would truly bring out the benefits of such predictions. The formation of DNA–platinum adducts and subsequent progression toward apoptotic cell death was believed to be the mechanism of action for platinum drugs. Recent advances have brought forth other novel mechanisms by which platinum drugs exert their cytotoxic effect. One such mechanism is the ability of platinum drugs to modulate the host immune system. Treatment regimen that includes platinum drugs in combination with immune checkpoint blockers, such as anti-CTLA4 antibody and ipilimumab, has been approved for metastatic melanoma, whereas anti-PD-L1 antibody, atezolizumab, has been approved for metastatic lung cancer (91).

Platinum drugs, although highly efficacious in patients carrying BRCA1 and BRCA2 mutations, come at a cost in the form of severe side effects—nephrotoxicty and ototoxicity being two of the major unwanted effects of cisplatin treatment. The major challenge is to target the drug specifically to the tumor alone and minimize its accumulation in non-tumor tissue. Development of better drug-delivery vehicles that can ensure targeted delivery at the tumor site will not only maximize availability of the therapeutic agent at the site of the tumor but also reduce accumulation in healthier tissue, thereby minimizing toxicity. Thus, a therapeutic regimen that includes inhibitors for the DNA repair pathway, a tumor-specific platinum drug, together with radiotherapy may prove to be an effective way of treating and managing the cancer burden due to BRCA1 and BRCA2 mutations.

## Author Contributions

MR was involved in the conceptualization of the topic and supervised the writing of the manuscript. SM wrote the manuscript and drew the figures. AD critically reviewed the article.

## Conflict of Interest Statement

The authors declare that the research was conducted in the absence of any commercial or financial relationships that could be construed as a potential conflict of interest. SM and MR are employees of Invictus Oncology Pvt. Ltd. and hold equity of IOPL. All remaining authors have no potential conflicts to disclose.
